# Deconstructing deployment of the innate immune lymphocyte army for barrier homeostasis and protection

**DOI:** 10.1111/imr.12709

**Published:** 2018-10-07

**Authors:** Francisca F. Almeida, Nicolas Jacquelot, Gabrielle T. Belz

**Affiliations:** ^1^ Division of Molecular Immunology Walter and Eliza Hall Institute of Medical Research Melbourne Victoria Australia; ^2^ Department of Medical Biology University of Melbourne Melbourne Victoria Australia

**Keywords:** homeostasis, innate immunity, innate lymphoid cells, mucosal immunity, protection

## Abstract

The study of the immune system has shifted from a purely dichotomous separation between the innate and adaptive arms to one that is now highly complex and reshaping our ideas of how steady‐state health is assured. It is now clear that immune cells do not neatly fit into these two streams and immune homeostasis depends on continual dialogue between multiple lineages of the innate (including dendritic cells, innate lymphoid cells, and unconventional lymphocytes) and adaptive (T and B lymphocytes) arms together with a finely tuned synergy between the host and microbes which is essential to ensure immune homeostasis. Innate lymphoid cells are critical players in this new landscape. Here, we discuss recent studies that have elucidated in detail the development of ILCs from their earliest progenitors and examine factors that influence their identification and ability to drive immune homeostasis and long‐term immune protection.

## 1 INTRODUCTION

The world of innate immune cells has greatly expanded in recent years. Broadly it includes innate lymphoid cells (ILCs) together with an array of unconventional lymphocytes such as γδ T cells, CD1‐restricted NKT cells, and mucosal‐associated invariant T (MAIT) cells. ILCs are distinct from other newly described innate cells as they lack recombined antigen‐specific receptors characteristic of B and T lymphocytes and many of the phenotypic lineage markers that define other immune cell subsets. Indeed, ILCs are enriched when the two genes *Rag1* and *Rag2* that regulate recombination machinery, selection, and diversity in other lymphocytes, are deleted.^1^ Nevertheless, ILCs exhibit a number of features that are reminiscent of T cells implying that they may be the innate counterparts of adaptive lineages.^2^ ILCs have generally been regarded to be an almost exclusively tissue‐resident population found at the barrier surfaces such as the skin, lungs, and intestinal tract.^3^ New evidence now suggests that colonization of tissues, replenishment, and rapid dissemination of ILCs depends at least partly on the capacity of these cells to move around the body in response to pro‐inflammatory signals allowing them to fight infection and maintain immune homeostasis. Here, we discuss the specific transcriptional pathways that are essential to regulate the generation and maintenance of ILCs. We focus on how recent findings are reshaping our understanding of the complexity of homeostatic regulation at barrier surfaces forcing us to rebuild the rules by which we understand how the immune system operates.

## 2 INNATE LYMPHOID CELL SUBSETS

Innate lymphoid cells are a heterogeneous family of immune cells that have shed new light on the architecture of the immune response and our understanding of how immune protection is orchestrated. ILCs express germline‐encoded receptors that enables them to respond rapidly to stimuli. In many cases, precisely how these receptors work has been unclear as little is known about the ligands activating the receptors. Recent evidence, however, suggests that NKp46 can recognize the cognate ligand complement factor P,^4^ and NKp44 can recognize platelet‐derived growth factor (PDGF)‐DD produced by tumors,^5^ highlighting additional crucial roles in recognizing soluble tissue components, in addition to recognition of pathogen‐derived ligands^6^, ^7^, ^8^, ^9^ to protect against infections and to mediate tissue repair. This feature allows them to deliver front line defense against the continual assault on the body from both foreign and commensal organisms as well as antigens derived from food and environmental sources.

Although we have only recently been readily able to dissect the diversity of ILC populations due to their rarity, NK cells, and lymphoid tissue‐inducer (LTi) cells were discovered more than 30 years ago. This established their prototypical roles in tumor immunosurveillance (NK cells)^5^, ^10^ and in the formation of secondary lymphoid tissues (LTi cells)^11^, ^12^ during embryogenesis, respectively. Our understanding of this family has now greatly expanded with the discovery of new previously unrecognized members that have been classified into three main subsets: ILC1, ILC2, and ILC3s.^13^ These groupings are largely aligned with effector T cells and are based on their expression of transcription factors and cytokine profiles.

ILC1s predominantly produce IFN‐γ following stimulation. They are defined by the surface receptors NK1.1 and NKp46 (CD335) together with their lack of lineage specific markers (including CD3, CD4, CD8, CD19, CD11c, and transcription factor RORγt). This reveals a heterogeneous population that can be further separated into NK cells (which express CD49b, also known as DX5) and non‐NK ILC1s (which express CD49a or VLA‐1α). Both NK cells and ILC1 express the transcription factor T‐BET (encoded by *Tbx21*), but generally only NK cells express EOMES (encoded by *Eomesodermin*, also referred as T‐box brain protein 2). These factors are associated with IFN‐γ production and anti‐tumoral activities. NK cells and ILC1 also differ in their lifecycle as NK cells seem to continuously recirculate around the body while non‐NK ILC1s appear to reside mostly in tissues such as the liver. In addition, it is likely that the specific tissues inhabited by ILC1 significantly influence their phenotype and function. For example, it has been shown that salivary gland ILC1 are phenotypically distinct from liver ILC1 or from intraepithelial ILC1.^14^

ILC2s produce interleukin(IL)‐5, IL‐9 and IL‐13 together with tissue repair factors such as amphiregulin. They are defined by their expression of the surface markers ICOS (Inducible T cell costimulator), KLRG1 (Killer cell lectin‐like receptor subfamily G member 1), Sca1, ST2 (IL‐33R), CD25 (IL‐2Rα), and IL‐7R together with the transcription factors GATA3 (GATA binding protein 3) and nuclear receptor RORα (RAR‐related orphan receptor α).^15^ Some variability in the expression of ST2, KLRG1, and CD25 has been observed depending on the tissue location and stimulus,^16^ while in most tissues, ICOS is reliably expressed and indeed required for their survival and cytokine production. ILC2 are mainly involved in responses to allergic stimuli and parasites and are thus found at several sites throughout the body including the lungs, spleen, gut, liver, and skin.^3^, ^17^

ILC3 are characterized by the expression of RORγt and production of IL‐22 and/or IL‐17. They are found enriched in mucosal tissues such as the intestine and are demarcated into three distinct subpopulations by their expression of CD4 and the NK receptor, NKp46 (encoded by *Ncr1*, natural cytotoxicity triggering receptor 1). LTi cells, which orchestrate the generation of lymphoid tissues during fetal development, express the coreceptor CD4 together with the chemokine receptor CCR6, but lack NKp46 expression.^11^, ^12^ Two additional populations of ILC3 are defined by their lack of CD4 expression combined with their expression, or lack of, NKp46. Although the marker CCR6 has been used to divide ILC populations into “helper” and “cytotoxic” ILC populations,^18^ it does not always appear to show clearly definable subsets within the ILC3 population. NKp46^+^CCR6^−^ ILC3 correspond to the effector population that depends on the upregulation of T‐BET for their formation.^19^, ^20^ CCR6^+^ ILC3 have been shown to express Major Histocompatibility Class II expression and exhibit some antigen processing capacity. This feature allows them to limit the expansion of commensal bacteria‐responsive CD4^+^ T cells through activation induced cell death thus preventing subsequent intestinal disease and dysregulation of the microbiome.^21^

### 2.1 Development of early innate lymphoid progenitors in bone marrow

**Figure 1:**
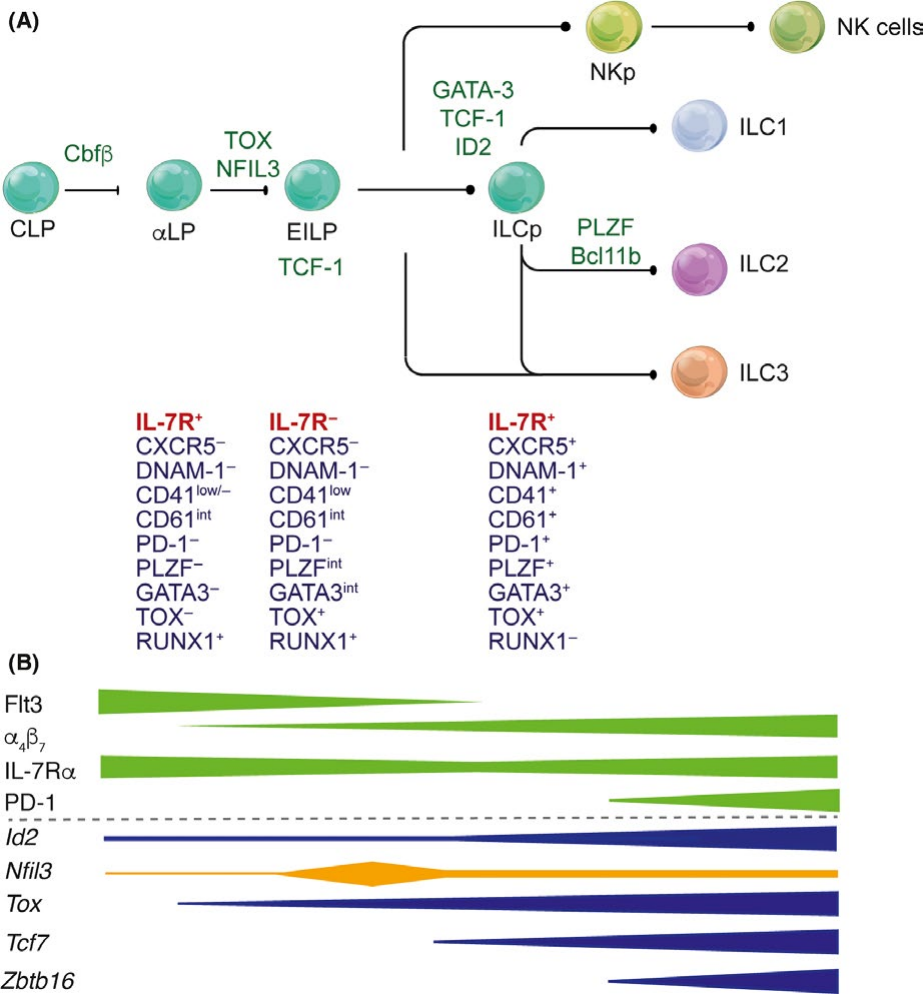
(A) Transcriptional regulation of ILC development from the common lymphoid progenitor (CLP) to mature ILC subsets 1, 2, and 3. It is now clear that the CLP transits through a series of intermediates including the early innate lymphoid progenitor (EILP) which in contrast with stages both preceding and following the EILP, downregulate the expression of IL‐7R. (B) Differential regulation of transcription factors and surface receptors is both dynamic and essential for diversification of ILC subsets

ILCs are thought to arise from all‐lymphoid progenitors (ALPs) which contains the common lymphoid progenitor (CLP) and the IL‐7Rα^+^ multipotent ILC progenitors.^18^, ^22^, ^23^, ^24^, ^25^ The major progenitor potential lies within the α_4_β_7_ fraction of the CLP.^26^ Although all ILCs derive from an IL‐7Rα^+^ progenitor, an additional stage, termed the early innate lymphoid progenitor (EILP) has recently been defined and notably is marked by the expression of the transcription factor T cell factor‐1 (TCF‐1, encoded by the gene *Tcf7*).^27^ *Tcf7* ^+^ progenitors expressed only low levels of IL‐7Rα, *Zbtb16* (also known as *Plzf*), and *Id2* (Inhibitor of DNA binding 2).^27^ What was distinct about this cell type was that it did not fit with the known linear progression of ILC differentiation that had been previously described. Distinct from other members of the progenitor network, the EILP did not express IL‐7Rα. This was perplexing but such a step in ILC differentiation could occur if EILPs did not arise from the ALP; or alternately, ILC progenitors could transition through a stage that depended on the downregulation and subsequent re‐expression of IL‐7Rα as normally occurs in developing thymocytes (Figure 1).^28^ Thus, the EILP would represent an intermediate developmental stage in which IL‐7Rα is transiently downregulated. Indeed, when the *IL7rCre* strain was crossed to a ROSA26‐YFP reporter strain and the *Tcf7* ^*EGFP*^ reporter, the temporal expression of *Tcf7* and IL‐7R amongst IL‐7R^+^ an IL‐7R^−^ cells could be ascertained.^29^ Indeed, it was then clear that the IL‐7R^−^ population carried the imprint of previous IL‐7R expression and that the EILP defines a critical step in ILC generation. Importantly, this work defined the link between the very early progenitor stages of the ALP and ILCP (ILC progenitor), and the EILP, and crucially pinpointed the requirement for differential regulation of receptor expression for this transition that may well have been normally overlooked (Figure 1).^29^ IL‐7R expression is therefore highly dynamic and tightly regulated by TCF‐1^30^ resulting in early expression in development, but subsequently downregulated to allow the EILP to give rise to ILCP.

### 2.2 The thymic pathway

Although ILCs in the adult typically originate from the bone marrow, emerging data points to an additional network that regulates thymic progenitors that are normally destined to establish T cell identity to adopt an innate fate. This possibility challenges the current paradigm but is plausible as ILC express many transcription factors that are characteristic of the T cell lineage including TCF‐1, GATA‐3, and Bcl11b together and signaling molecules ICOS, PD‐1, LTA, LCK, and ITK.^26^, ^30^, ^31^, ^32^, ^33^, ^34^ This shared transcriptional network suggests that the fate decisions of T cells and ILCs are inextricably linked. Providing substance to the possibility of a thymic ILC origin, Miyazaki et al^35^ showed that the E proteins E2A and HEB, which interact with DNA binding proteins (Id), suppress the induction of the ILC gene program by promoting T cell specific genes such as Notch receptors. This effect is executed by interfering with the level of ID2 expression, a transcription factor necessary for the development of all ILC and the maintenance of several mature subsets.^35^, ^36^ Overexpression of ID3, which can function similarly to ID2, promotes the generation of NK cells from thymocytes,^37^ while overexpression of ID1 in transgenic mice enhances the development of ILC2 in multiple organs, most notably in the thymus.^38^, ^39^ ID1 itself is not generally found in immune cells but ectopic expression of Id proteins, or the removal of their E protein binding partners, serves to reciprocally enhance their expression and drive ILC development. The thymus is not essential for the formation of ILC2 but they can be generated from thymic progenitors when they are cultured with IL‐7 and IL‐33^39^, ^40^ suggesting that the balance of innate and adaptive immune cell fate outcomes depends on the combination of transcription factors together with external stimuli encountered by cells. This effect has also been shown in vivo.^35^, ^41^ Similarly, deletion of transcription factors that normally define T cell identify, such as BCL11b, can have the capacity to derepress the dominant T cell developmental pathway in the thymus resulting in NK cells development.^42^ In addition, however, BCL11b can fine tune the balance between ILC2 and ILC3 in the periphery to act as a sensitive rheostat for ILC subset development in response to stimuli.^43^, ^44^ Within the thymus, maintaining the delicate balance of ILC subsets appears to be crucial for the integrity of the thymus and the emergency generation of ILC. Indeed, while intrathymic ILC3 play a critical role in thymic regeneration where they produce abundant IL‐22 to drive thymic repair following graft‐versus‐host disease,^45^, ^46^ ILC2 have recently been shown to be the dominant ILC population within the thymus after birth.^47^ The accumulation of ILC2 in the thymus over time raises the possibility that type 2 cytokines produced by these cells play a role in supporting normal thymic function. However, the exact origin of both of these subsets is yet to be explored.^48^, ^49^

Collectively, these findings challenge the notion that the bone marrow is the only source of ILCs in the adult and instead raises the idea that an evolutionary mechanism has arisen providing multiple pathways to generate ILCs to protect the body against insults.

### 2.3 Key drivers of ILC subset differentiation

A core group of transcription factors are essential for the early development of ILCs. These include Inhibitor of DNA binding 2 (ID2, *Idb2*), T cell factor 1 (TCF‐1, encoded by *Tcf7*), Nuclear factor interleukin‐3 (NFIL3, *E4 bp4*), Thymocyte selection associated high mobility group box (TOX, *Tox*), and GATA binding protein 3 (GATA3, *Gata3*). These factors collaborate to orchestrate the sequential restriction of progenitors into individual ILC lineages and have been described in detail elsewhere.^50^, ^51^, ^52^, ^53^, ^54^

#### 2.3.1 NK cells

**Figure 2:**
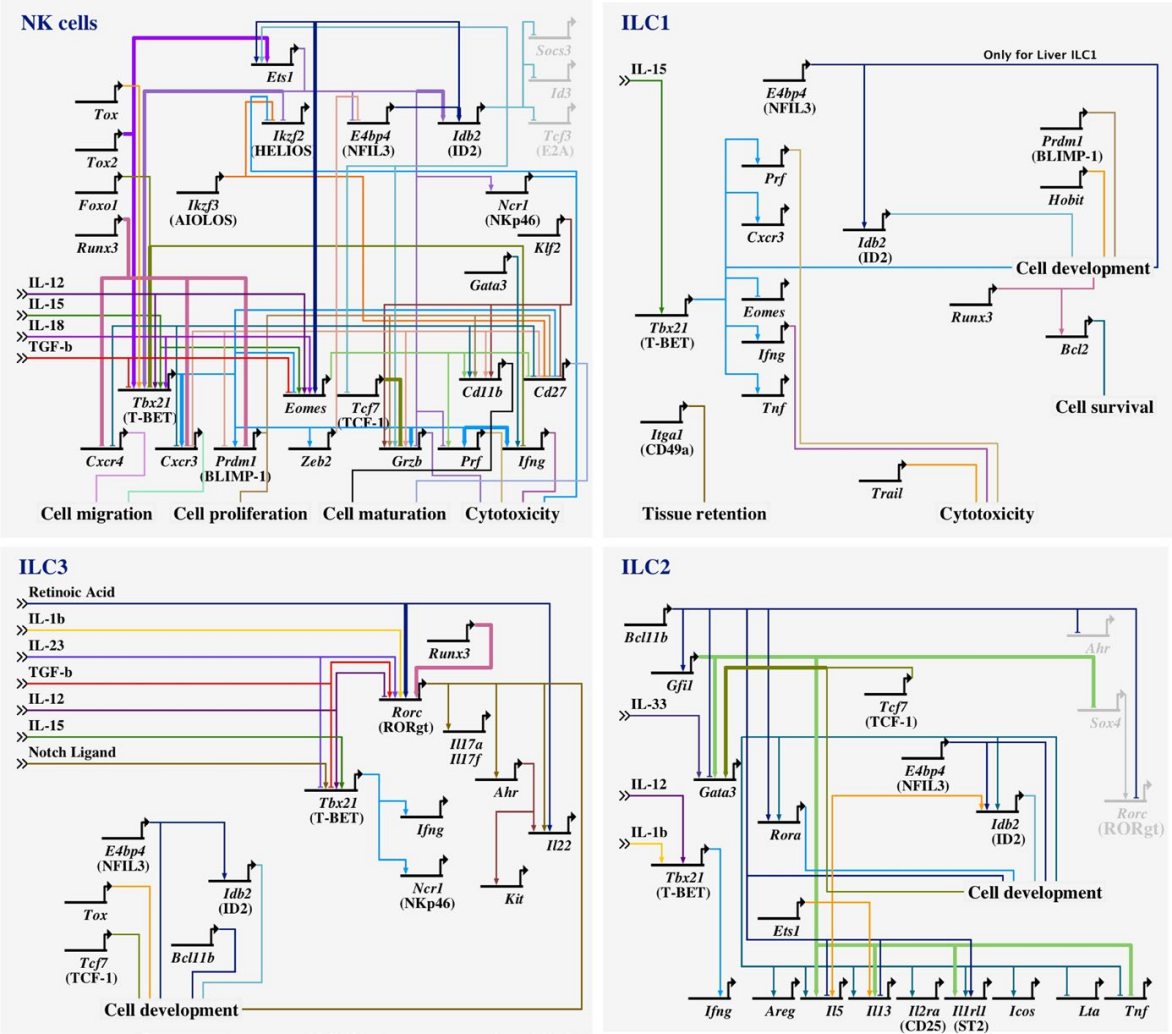
Gene regulatory network illustrating the involvement of key transcription factors in NK cells, ILC1, ILC2, and ILC3 development using BioTapestry software (Version 7.1.1m, biotapestry.org). Critical extracellular signals influencing ILC functions and mediating ILC plasticity are depicted. Networks were designed based on ChIP data or reporter assays^61^, ^79^, ^91^, ^92^, ^102^, ^176^, ^177^, ^178^, ^179^, ^180^ (thick lines) together with gene deletion or overexpression systems (regular lines).^18^, ^19^, ^40^, ^43^, ^44^, ^57^, ^58^, ^59^, ^60^, ^61^, ^64^, ^72^, ^74^, ^75^, ^78^, ^79^, ^85^, ^86^, ^87^, ^89^, ^90^, ^91^, ^92^, ^93^, ^95^, ^98^, ^110^, ^118^, ^128^, ^169^, ^174^, ^176^, ^177^, ^178^, ^179^, ^180^, ^182^, ^183^, ^184^, ^185^, ^186^, ^187^ Nonexpressed genes are depicted in gray. Linkages are color‐coded for clarity only. The individual genes are shown in schematic form only. The lines indicate the direct binding of the protein encoded by the indicated gene to the regulatory regions of the linked target genes, which leads to transcriptional activation or repression. *Runx3*, runt related transcription factor 3; *Ets1*, ETS proto‐oncogene 1; *Ikzf2* (HELIOS), IKAROS family zinc finger 2; *Ikzf3* (AILOS), IKAROS family zinc finger 3; *Nfil3*, nuclear factor, interleukin 3 regulated; *Idb2*, inhibitor of DNA binding 2; *Socs3*, suppressor of cytokine signaling 3; *Id3*, inhibitor of DNA binding 3; *Tcf3* (E2A), transcription factor 3; *Prf1*, Perforin 1; *Gzmb*, granzyme B; *Tcf7*, transcription factor 7; *Zeb2*, zinc finger E‐box‐binding homeobox 2; *Prdm1* (BLIMP1), PR domain zinc finger protein 1; *Foxo1*, forkhead box protein O1; *Tox*, thymocyte selection associated high mobility group box; *Gata3*, GATA binding protein 3; *Itga1*, integrin subunit alpha 1; *Ifng*, interferon‐gamma; *Areg*, amphiregulin; *Il2ra*, interleukin‐2 receptor subunit alpha; *Icos*, inducible T cell costimulator; *Lta*, lymphotoxin alpha; *Tnf*, tumor necrosis factor; *Sox4*, SRY‐Box 4; *Rorc*, RAR related orphan receptor C; *Rora*, RAR related orphan receptor A; *Gfi1*, growth factor independent 1; *Bcl11b*, B cell CLL/lymphoma 11B; *Ahr*, aryl hydrocarbon receptor. CD, cluster of differentiation

Several NK cell progenitors have been identified to give rise to different peripheral NK cell subsets. NK cells are guided through progressive developmental stages by the expression of specific transcription factors necessary for NK lineage commitment and maturation. The pre‐pro NK cell precursor population is the earliest identified committed NK cell progenitor that shows a highly enriched capacity to generate NK cells.^55^ These cells express the IL‐2Rβ chain (CD122), Sca‐1, IL‐7Rα, and ID2 but lacked markers typically expressed on fully differentiated mature NK cells such as NK1.1, NKp46, and CD49b.^55^ In the bone marrow, this progenitor further committed into NK progenitor (NKP)^55^, ^56^ and subsequently into immature and mature NK cells under the influence of a core transcription program including but not restricted to *Id2*,^55^ *Gata3*,^57^ *Nfil3*,^58^ *Klf2*,^59^ *Eomes*,^60^ *Tbx21*,^61^ *Tcf7*,^27^ *Tox,* 62 and *Ets‐1* (Figure 2).^63^ Interestingly, NFIL3 expression is essential for the development of all ILC subsets^64^ but appears to be only required early and transiently to implement the ILC program as *Nfil3* deletion in ID2^+^ mature NK cells does not affect NK cell survival or function.^26^, ^50^, ^65^

Immature and mature NK cells are present in peripheral tissues and blood and are characterized by the expression of NK1.1 and NKp46. CD11b and CD27 expression subdivides NK cells into immature (Imm, CD11b^−/low^CD27^+^), mature 1 (M1, CD11b^+^CD27^+^) and mature 2 (M2, CD11b^+^CD27^−^) subsets.^66^ These mature subsets may express other surface molecules, such as KLRG1,^67^ CD62L,^68^ and DNAM‐1^69^ that further characterize their specific phenotype and functions. In parallel with the progressive change in surface molecule expression during maturation, NK cell function is also affected. Most strikingly, mature NK cells become less proliferative and produce less cytokine but conversely, they gain cytotoxic function as they further mature from M1 into M2 populations.^70^

#### 2.3.2 ILC1

ILC1 originate from the innate lymphoid cell progenitor (ILCP) which also gives rise to other subsets of ILCs such as ILC2 and some ILC3.^71^ ILC1 express NK1.1 and NKp46 but are distinct from NK cells. They generally lack CD49b or EOMES expression but depend strongly on T‐bet expression^72^ in contrast with splenic NK cells which only partly rely on this factor.^18^, ^73^, ^74^ In addition, while BLIMP1 is required to fully upregulate T‐BET expression in splenic NK cells, genetic deletion does not result in a marked defect in NK cell populations.^75^ Nevertheless, an unexpected synergy between BLIMP1 and HOBIT (homolog of BLIMP1 in T cells or ZNF683), a transcription factor normally controls tissue‐residency in CD8^+^ T cells, is essential for their development (Figure 2).^12^, ^64^, ^65^, ^74^, ^76^, ^77^, ^78^ ILC1 also preferentially express other surface molecules including IL‐7Rα, TRAIL (Tumor necrosis factor apoptosis‐inducing ligand), and CD49a (also known as integrin alpha‐1) and their survival is strongly regulated by the transcription factor RUNX3.^79^ TRAIL expression is dictated by signaling though the NKp46 receptor.^80^, ^81^, ^82^ In certain inflammatory conditions, NK cells and ILC3 are converted into ILC1‐like cells (sometimes referred to as ex‐NK cells and ex‐ILC3 respectively) and downregulate EOMES or RORγt expression in favor of T‐BET and TRAIL expression and exhibit enhanced IFN‐γ production.^83^, ^84^ ILC2 stimulated with IL‐1β potentiating IL‐12 responsiveness, or IL‐12 itself, also acts as a potent driver for this subset to acquire features of ILC1 cells.^85^, ^86^, ^87^, ^88^ Thus, in inflammatory settings each subset appears to be able to reprogram its capability to become IFN‐γ‐producing cells with potent effector functions.

#### 2.3.3 ILC2

The development of ILC2 is guided by the core transcriptional regulators RORα,^40^ GATA3,^22^, ^89^ TCF‐1,^90^, ^91^ Gfi1,^92^ and Bcl11b (Figure 2).^43^, ^93^ ILC2 are typically characterized by their expression of ST2 (IL‐33R), ICOS, and GATA3. GATA3 is expressed by ILC subsets and their progenitors and is thus required for their development. ILC2 express GATA3 at high levels in mature cells while ILC3 depend on sustained GATA3 expression to maintain NKp46^+^ identity and IL‐22 production by repressing the ILC3 LTi cell program.^94^, ^95^ A number of different ILC2 subsets are now recognized, including (a) natural ILC2 (nILC2, ST2^+^Thy1^high^KLRG1^intermediate^IL‐17RB^low/−^) and (b) inflammatory ILC2 (iILC2, ST2^−^Thy1^low^KLRG1^high^IL‐17RB^+^) which are distinguished by their level of expression of the Killer Cell Lectin Like Receptor G1 (KLRG1).^14^ iILC2 are induced to undergo significant proliferation in response to IL‐25 while IL‐33 can drive some expansion of nILC2^14^ and play an important role in the egress of ILC from the bone marrow through regulation of CXCR4.^96^ Programmed cell death program 1 (PD‐1, or CD279) signaling has recently been shown to negatively regulate KLRG1^+^ ILC2 limiting their capacity to inadvertently expand and induce pathology.^97^ Although ST2 has typically been used to identify ILC2, iILC2, in contrast with nILC2, do not express ST2 and thus this receptor cannot be universally used to identify ILC2 across different tissues or under different conditions of inflammation.

#### 2.3.4 ILC3

RORγt is the cornerstone transcription factor identified as essential for the development of ILC3^98^ and opened the door to the identification of the three major different subsets of ILC3 (Figure 2). The expression of the T cell co‐receptor CD4 distinguishes the prototypic CD4‐expressing ILC3, LTi cells, and CD4 negative populations. LTi act to orchestrate the generation of nascent lymphoid tissue initiated by the interaction between LTi cells and stromal organizer cells and that is dependent upon LTi‐expressed lymphotoxin.^99^, ^100^ CD4^−^ ILC3 are distinguished by the expression of the natural cytotoxicity receptor (NCR), NKp46, resulting in NKp46^+^ and NKp46^−^ ILC3 subsets. The transition between NKp46^−^ and NKp46^+^ ILC3 depends on induction of T‐BET via NOTCH2 interactions^19^ and this could be regulated by the strength of the inflammatory stimulus in the environment.^20^ These two cornerstone studies opened the way to begin to tease apart the precise machinery that guides the development of ILC3, particularly NCR^+^ ILC3, revealing that a number of transcription factors are essential for the development of these cells. These include the aryl hydrocarbon receptor (AHR) which is regulated in ILC3 by RUNX3^79^ and is sensitive to signals derived from the microbiota and dietary components that generate aryl hydrocarbons.^101^, ^102^, ^103^, ^104^

### 2.4 The complexity of the NK cell and ILC1 subsets and consequences of disruption of NKp46 signaling pathway

**Figure 3:**
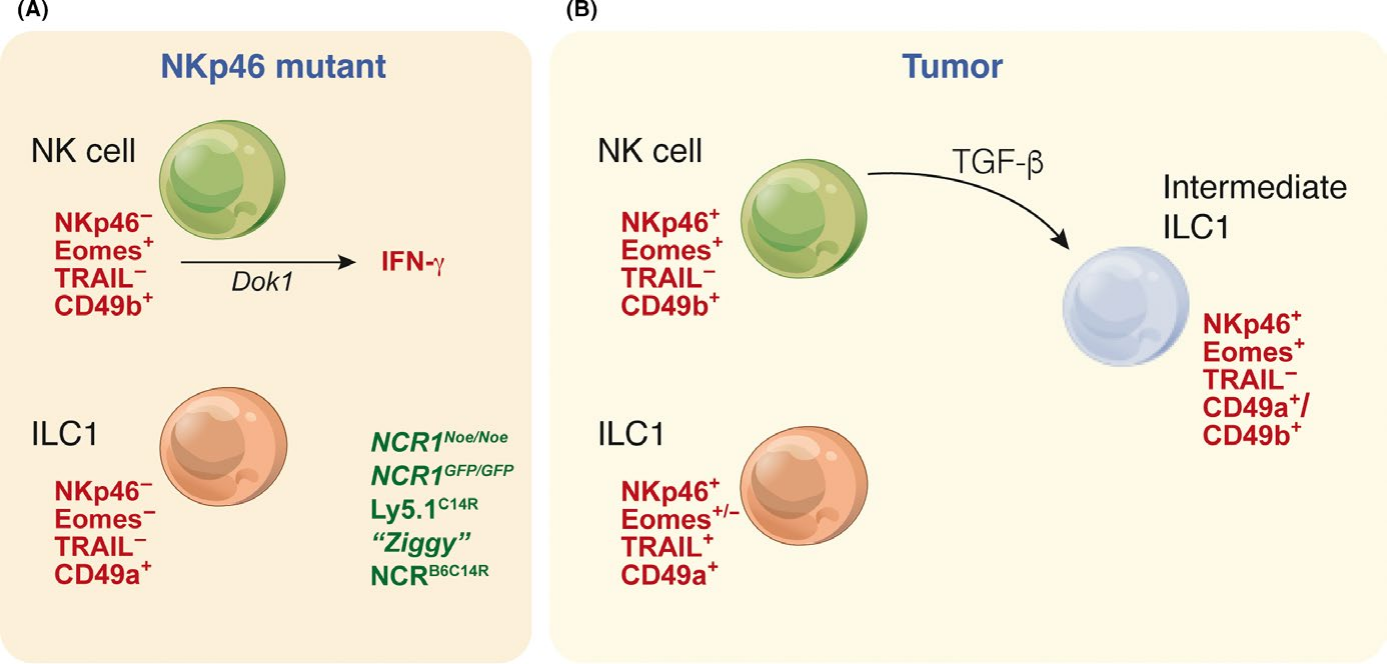
NK cells and ILC1 exhibit transitional phenotypes and functional alterations in response to tumors. (A) Multiple models have now been identified that highlight that deleted or unstable NKp46 results in ablation of TRAIL expression and impaired anti‐tumor activity. (B) Distinct NK cell and ILC1 subsets exist at steady‐state but under the influence of TGF‐β, tumor ILC1 acquire the expression of EOMES and surface markers such as CD49b normally expressed by NK cells

Our ability to ascertain the functions of NK cells has relied heavily on their identification as NK1.1^+^ cells. NK1.1^+^ cells have been attributed the important defense mechanism of immunesurveillance protecting from the emergence of cancer.^105^, ^106^, ^107^ With the discovery of ILC subsets and tracking of cells using the NKp46 receptor in combination with transcriptional regulators, it has become clear that the classical NK cell compartment contained not one, but two subsets of cells—ILC1 and NK cells. Thus, it could no longer be assumed that all the functions credited to NK cells were in fact due to NK cells alone but instead may reflect the outcome of a mixed population of cells that also contained ILC1. Further complicating the interpretation of the data attributable to individual subsets was the emergence of plasticity between a number of ILC subsets implying that alternate flexible programs that did not neatly fit into the subset classification could be identified. In an unexpected twist, recently three groups identified a point mutation in the *Ncr1* gene in the congenic CD45.1^+^ mice used for many studies of lymphocyte tracking and function analysis (Figure 3A, Table 1).^80^, ^81^, ^82^ These lines, all derived from Jackson Laboratories, exhibited a single amino acid mutation from cysteine to arginine at amino acid 14 (C14R) which is localized in the region of the signal peptide of *Ncr1*. This mutation did not alter the overall expression of *Ncr1* mRNA but significantly impaired the surface expression of NKp46 through failed trafficking within the cell.^80^, ^81^, ^82^ These findings have major implications for previous studies of NK cells and ILC1 where these mice have been used, even further complicating our interpretation of earlier data (Table 1).

**Table 1:** NKp46 regulation of ILC function

Genetic modification	Phenotype		References
Mutation			
Ly5.1^C14R^ (C14R)	Loss of surface NKp46 expression Loss of TRAIL expression Normal NK cell and ILC1 numbers Altered NK cell maturation	Resistance to MCMV viral infection Reduced Dok‐1 augments IFN‐γ expression Complete loss of surface NKp46 expression	Jang et al^82^
*‘Ziggy’* (C14R)		Reduced cytotoxicity by ILC1	Turchinovich et al^80^
Ly5.1^C14R^ (C14R) (+ >300 additional genes)		Susceptibility to tumor	Almeida et al^81^
NCR^B6C14R^ (C14R)		Unstable NKp46 surface expression	Almeida et al^81^
*Ncr1* ^*No*é*/No*é^ (W32R)	Loss of surface NKp46 expression	Hyperactive NK cells Increased IFN‐γ production NKp46 retention in endoplasmic reticulum Susceptibility to tumor Increased Helios expression	Narni‐Mancinelli et al^110^
*Ncr1* ^*No*é*/No*é^ (W32R)	Unstable surface NKp46 expression	Altered glycosylation of NKp46 NKp46 retention in endoplasmic reticulum	Glasner et al^111^, ^188^
Deletion			
*NCR* ^*gfp/gfp*^	Loss of intracellular and surface NKp46 expression	Susceptibility to influenza virus infection	Gazit et al^108^
*NCR* ^*gfp/gfp*^		Loss of ILC1 (not observed in^81^, ^113^)	Wang et al^112^
*NCR* ^*gfp/gfp*^		Reduced IFN‐γ Loss of tumor control and fibronectin 1‐driven remodeling	Glasner et al^189^
*NCR1* ^*iCre/iCre*^	Severe impairment of NKp46 expression	Normal NK cell numbers and function	Narni‐Mancinelli et al^109^

The mutant mice, referred to as Ly5.1^C14R^, showed normal numbers of NK cells and ILC1 but a modest alteration to the immature and mature NK cell subsets.^81^ To date, several models affecting *Ncr1* expression have been generated. These include the *Ncr1* ^*gfp/gfp*^ *,* 108 *Ncr1* ^*iCre/iCre*^ *,* 109 and *Ncr1* ^*Noé/Noé*^ 110 mice. The *Ncr1* ^*gfp/gfp*^ mice have a complete loss of NKp46 expression, while *Ncr1* ^*Noé/Noé*^ mice exhibit a mutation at W32R^110^ which affects surface expression of NKp46.^110^, ^111^ A recent study also suggests that ILC1 development is intrinsically dependent on NKp46^112^ but this work contrasts with other studies that did not observe such effect.^81^, ^113^ Thus, the exact consequences of *Ncr1* loss requires deeper investigation to determine precisely how it affects ILC1 and NK cell function. Nevertheless, the loss of stable expression of NKp46 on the surface of cells has been shown to be broadly important and crucial for protection from influenza virus^82^ and tumor control^81^ but paradoxically appears to confer higher resistance in MCMV infection.^82^ In some cases, such as influenza, the hemagglutinin and neurominidase viral proteins have been purported as endogenous ligands^6^, ^108^, ^114^ while B16F10 melanoma cells are known to be controlled by NK cells.^106^, ^115^ However, the resistance to MCMV infection identified in this study was a surprise and was correlated with enhanced IFN‐γ expression.^82^ Increased IFN‐γ production was attributed to the reduction in expression of the gene *Dok1* which has previously been proposed to augment IFN‐γ.^116^ However, enhanced IFN‐γ expression was not observed in all studies of mice carrying the C14R mutation.^81^ Using exome sequencing, more than 300 genes were found to differ between Ly5.1^C14R^ mice and their Ly5.2 counterparts.^81^ It is therefore likely that many associations between NKp46 expression and function will emerge and that the newly developed NCR^B6C14R^ mouse, generated on an C57BL/6 background and carrying only the NCR mutation without disruption of other genes, will be an important tool in future studies.^81^ Despite this, the total loss of NKp46 expression, or unstable expression, was associated with the loss of TRAIL in both NK cells and ILC1.^80^, ^81^, ^113^ Similar to NKp46, TRAIL was transcribed but was unable to migrate to the cell surface in the absence of NKp46.^113^ This might occur if NKp46 and TRAIL comprise a single protein complex in cytoplasmic vesicles although the mechanism of release is not yet known. Collectively, these studies highlight the confounding nature of some earlier studies and the necessity to systematically ascertain the roles of NK cells and ILC1 in models where genes affecting function are not unknowingly disrupted.

### 2.5 ILC plasticity and the common default pathway

The broad subsets of ILCs have, through the development of elegant and novel tools and vigorous investigation, been relatively well‐elucidated. However, many questions remain around the programs that define each subpopulation, as well as the cellular and molecular triggers that allow so called “plasticity,” or the capacity to adopt a different phenotype. This attribute potentially enables the different ILC subsets unprecedented flexibility to respond to the encountered stimuli. Key transcription factors define the fundamental lineage fate program adopted by early progenitors. RORγt^+^ ILC3 were the first subset in which transformation into a cell type with characteristics mirroring those of ILC1 was identified, establishing this phenotype in response to pro‐inflammatory stimulation^83^. These cells, known as ex‐ILC3, carried the historical imprint of RORγt expression but become capable of producing IFN‐γ. Subsequently, it has been discovered that when ILC3 or ILC2 are exposed to a combination of stimuli, including IL‐1β, IL‐12, and IL‐33 for ILC2 (to generate ex‐ILC2) or IL‐12 in the case of ILC3, inflammatory pathways and T‐BET expression are activated, enabling the production of IFN‐γ.^86^, ^87^, ^88^ These stimuli reprogram both the phenotypic identity and function of ILC subsets such that they could now masquerade as ILC1‐like cells that potentially drive immune pathology. Transforming growth factor (TGF)‐β was shown to be a significant driver of the conversion of NK cells into intermediate NK‐ILC1 and ILC1 cells (Figure 3B).^84^, ^117^ TGF‐β accumulation often occurs in tumors in which it drives immunosuppression. Under TGF‐β stimulation, anti‐tumor NK cells are converted into intermediate NK‐ILC1 and ILC1 cells that are unable to control tumor growth and metastases formation.^84^, ^117^

Within ILC3 subsets, T‐BET also drives the development of NCR^+^ ILC3^19^, ^20^ but this appears not to be a terminal end state for these cells as using fluorescent fate‐mapping (YFP), both YFP^+^ NCR^+^ and NCR^−^ ILC3 have been detected suggesting that NCR^+^ ILC3 can revert to the NCR^−^ phenotype.^118^ Similar to ILC1, TGF‐β is also a key driver of the formation of NCR^+^ ILC3.^118^ Why such an interconversion might exist is not yet completely clear but it could act as a brake on the formation of this highly reactive effector subset as an inflammatory stimulus subsides. This would limit immunopathology that may occur as a sequel to a prolonged or uncontrolled response.^118^ NCR^−^ ILC3 are intriguing—they share many features with NCR^+^ ILC3 such as their capacity to constitutively produce IL‐22 but are even more closely related to LTi cells. Although NCR^−^ and NCR^+^ ILC3 differ by several hundred genes, only a handful of genes are differentially expressed between LTi cells and NCR^−^ ILC3 making them almost indistinguishable despite that LTi cells are derived from a fetal liver progenitors while NCR^−^ ILC3 arise from a bone marrow progenitor.^118^, ^119^, ^120^ Similarly, NCR^+^ ILC3 have been mapped to express different levels of the transcription factor RORγt (dissecting high and intermediate expressing populations) and these populations differed by fewer than 100 genes. Again, these subpopulations appear to be phenotypically distinct, but why they would share such closely aligned gene signatures is not clear.^118^

## 3 IMMUNE HOMEOSTASIS AT MUCOSAL SURFACES: ILC NETWORKS IN THE GUT

The mucosa is colonized by the bulk of immune cells found in the body. These cells sense information from intestinal contents such as the trillions of microbes that inhabit the gut and food components. This landscape poses considerable challenges to maintain health. To that end, the immune system is charged with the task of balancing responses to maintain mucosal homeostasis. Fending off invading pathogens is clearly important, but maintaining immune homeostasis at these highly vulnerable surfaces is perhaps the single most important function that prevents succumbing to disease. In both the gut and the lung, the epithelium physically separates microbes from the immune cells but a constant dialogue between these compartments drives the integration of signals that guides homeostasis. For example, in addition to physical interactions between microbes and immune cells, it has been uncovered that metabolites generated by microbes provide essential signals to immune cells in the host‐microbiota homeostatic network.

### 3.1 Maintaining ILC at mucosal surfaces

If continuous protection is to be afforded by ILCs, then it is necessary for these cells to position themselves, and regenerate, at mucosal surfaces despite the pressures exerted by exposure to constant insults. The development and maintenance of this protective shield depends on two features. First, the provision of survival factors such as cytokines within the local tissue microenvironment. Second, the deposition of ILC at mucosal sites.

The cytokine IL‐7 is essential for the development of ILCs, particularly ILC3, which are severely reduced in IL‐7‐deficient mice.^121^ IL‐7 stimulation is the key to drive proliferation and survival of ILC3 but it also plays a role in preserving ILC2 and ILC3 numbers to maintain lymph node size.^122^ ILC3 also play a key role, distinct from the effects on stromal cell^123^ and dendritic cell^124^ number which also affect lymph node size, in gating transit of immune cells into lymph nodes via high endothelial venules. This contrasts markedly with ILC2 which have no apparent impact on this gating function.^122^ Although IL‐7 is integral to ILCs, IL‐7‐independent pathways that support ILC survival also exist. This includes IL‐15 which has well‐described roles in the development of NK cells^125^, ^126^ and ILC1^73^ but can drive a separate program to at least partially support ILC2 and ILC3 in the intestine.^127^ Responsiveness, however, is modulated by sensitivity to IL‐15 which differs amongst the subsets in the gut. Intestinal NK1.1^+^ cells expand readily in response to IL‐15 but RORγt^+^ NKp46^+^ ILC3 are unaffected.^121^ In NK cells, sustained responsiveness to IL‐15 is maintained by the continuous expression of the transcription factor ID2.^128^ Whether ID2 plays a similar role in other ILC subsets in establishing differential responsiveness is not yet clear. In addition to IL‐7 and IL‐15, that regulate ILC maintenance at steady‐state, IL‐2 can also play a role in supporting ILC2, ILC3 and tuning NK cell sensitivity.^129^, ^130^ IL‐2 availability often depends on efficient local competition for supplies by ILC from regulatory CD4^+^ T cells, which strongly influence the outcome of local interactions.

It has become clear that cytokine maintenance of ILCs depends on complex interactions with specialized epithelial cells that are found in the gut and lungs. In the intestine, the number of ILC2 and ILC3 are delicately and reciprocally balanced. The gut epithelium is complex and composed of well‐known absorptive enterocytes, goblet cells, and Paneth cells found in the crypts, while other cells are rather less frequent and much more poorly characterized or understood. This includes the chemosensory epithelial Tuft (or “brush”) cells in the epithelial lining of the intestines. Their development is regulated by the transcription factor Pou domain, class 2, transcription factor 3, *Pou2f3*, and they play a central role in triggering the induction of type 2 immune responses following parasite infection. IL‐25 is essential to drive the amplification of ILC2^131^ but until recently, the exact cell type that produced this cytokine was unknown. Analyses of intestinal epithelial cells revealed that only a very small proportion of cells, the Tuft cells, produced IL‐25.^132^, ^133^, ^134^ Establishing this exclusivity was facilitated by generation of an IL‐25 reporter mouse line. This mouse also revealed that Tuft cells were not the source of other important epithelial cytokines such as IL‐33 and thymic stromal lymphopoietin (TSLP) that can also activate ILC2.^35^ Elucidation of this pathway is exciting and prompts us to ask whether other novel cell types found in the intestine, which as yet relatively poorly characterized, might also contribute to maintaining the ILC network and the elegant cooperation between epithelial and immune cells that drives homeostatic balance between ILC2 and ILC3.

### 3.2 Tissue residency and circulation

**Figure 4:**
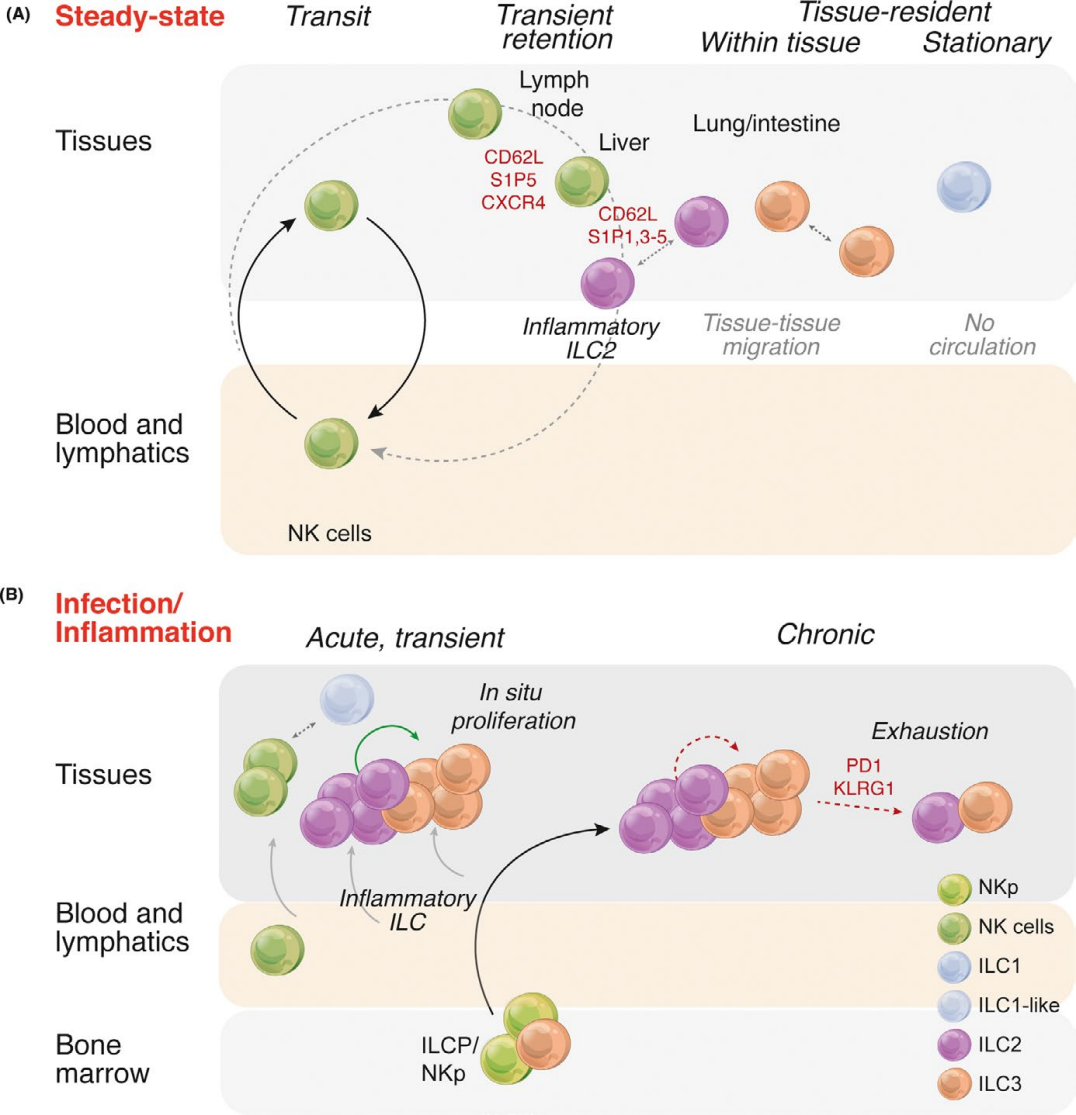
Regulation of ILCs at (A) steady‐state and during (B) infection and inflammation. (A) It is emerging that ILCs express a range of surface receptors that allow fine tuning of the positioning of different populations in tissues or between tissues dispelling the notion that ILCs are entirely sedentary within tissues. NK cells remain the most mobile with the capacity to move freely in the blood engaged in immunosurveillance, or to be recruited into tissues by modulating receptor expression. Only ILC1 appear to exhibit a truly tissue‐resident existence. (B) A proposed model for the replenishment of ILC in tissues. At steady‐state, slow proliferation of ILC within the tissues themselves allows the balance of subsets to be maintained. In an acute transient infection this may also be the case and that any temporary depletion would be rapidly replaced through enhanced local proliferation. If inflammation continues this might result in depletion that is not readily overcome by local proliferation and could lead to a state of “exhaustion” akin to that exhibited by T cells. Alternately, inflammation may drive differentiation of bone marrow progenitors and export in the blood to the affected tissues

Except for NK cells which are mostly circulating,^135^, ^136^ ILCs have generally been thought to be largely restricted to the tissues in which they are found, having established their niche early in ontogeny.^137^, ^138^ This view of ILCs is predicated on several pieces of evidence including (a) ILCs are poorly replaced following transplantation,^137^, ^139^ (b) mature ILCs do not appear to exchange between mice in which the circulatory system is conjoined in models of parabiosis,^137^ and (c) few ILC that express a mature phenotype are found in the bone marrow.^18^, ^71^ In mice ILC replacement is extremely poor following exposure to lethal doses of γ‐irradiation,^137^ and in patients who display mutations in the common γ chain cytokine receptor subunit IL‐2Rγ, or the tyrosine kinase JAK3, tissue ILCs fail to be effectively reconstituted.^139^ In part, this has been attributed to the extremely slow turnover of ILCs.^140^ Taken together, these findings have implied that ILCs are fundamentally sessile and are not readily replaced by bone marrow‐derived cells. They are thus proposed to be replenished predominantly by local expansion within peripheral tissues in response to various stimuli.^137^ This model prevails if it is assumed that (a) bone marrow‐resident progenitors would generate mature ILC within the bone marrow itself, (b) circulating ILC exhibit the same phenotype, receptor and transcription factor expression as characterized for mature tissue‐resident ILC and (c) that homing receptor expression is static rather than dynamically regulated in response to the many cues to which ILCs are exposed. However, mounting evidence suggests that both local movements of ILCs can occur to “concentrate” them in an area (eg, ILC3 accumulate in perifollicular areas of Peyer's Patch and intestinal crypts^141^, ^142^), as well as more generalized movements required for effector cell distribution and immunosurveillance within the body (Figure 4). Thus, it remains to be determined whether ILCs actually exhibit life‐long tissue residency and fail to move in response to infection or insults.

ILCs are no doubt more radioresistant than a number of other immune cell lineages such T cells, B cells and most dendritic cells. This feature appears to be protective. In the gut, irradiation induces rapidly proliferating epithelial cells in the intestinal crypts to undergo apoptosis and then to be regenerated from Lgr5^+^ intestinal stem cells.^143^, ^144^ Radioresistant ILC3 protect the intestinal stem cell pool via their secretion of IL‐22.^145^ Complete loss of IL‐22 results in increased pathology in the gut, a severe loss of intestinal stem cells and significantly reduced survival.^145^ IL‐22 secreted by radioresistant ILC3 also drives thymic regeneration following irradiation which induces significant damage across thymocytes and thymic epithelial cells.^45^ Thus, radioresistance is an important attribute of the ILC family which has important implications in tissue regeneration and protection against graft‐versus‐host disease following bone marrow transplantation.

Recent studies indicate that circulating ILCs display their own unique molecular program. This appears to be distinct from ILC phenotypes that have been previously described or that might be predicted.^146^ Although CD117^+^ ILCP were shown by single cell sequencing to express transcripts for genes known to control ILC differentiation, they lacked a number of signature genes such as *TBX21*,* GATA3,* and *RORC* and cytokines including IL‐5, ‐13, ‐17, ‐22, and IFN‐γ found in mature cells. This study highlights that circulating ILC progenitor cells occur in the blood to enable seeding and establishment of ILCs in more distant tissues sites. Although it is purported that the major role of these cells is early in development, it provides a labile population responsive to triggering from inflammatory stimuli that could afford rapid repopulation of tissues. In the case of HIV, irreparable loss of ILCs, mainly ILC3, has been shown to occur but this arose from depletion of circulating ILC progenitors and under conditions of chronic stimulation, neither local or circulating ILC stores were capable of rebuilding the integrity of the tissues and mucosal protective barrier.^147^ Furthermore, other ILC subsets have been shown to express receptors that undoubtedly drive their entry into both the lymphatic and blood systems creating a pathway for their distribution to other tissue sites. For example, inflammatory ILC2 are sensitive to sphingosine 1‐phosphate(S1P)‐medicated chemotaxis during anti‐helminth immunity.^148^ Thus, evidence is beginning to emerge that suggests in some situations at least, ILCs can be mobilized and deployed to tissues to ensure mucosal protection (Figure 4).

NK cells have been typically regarded at undergoing continual recirculation in the blood, a feature essential to mediate immunosurveillance allowing rapid and potent responses to tumors. However, NK cells also express a variety of chemokine receptors and can be provoked to migrate in response to factors that do not belong to the chemokine superfamily. These include the proinflammatory protein chemerin^149^ and the G‐protein coupled receptor S1P_5_ 150 molecule that can affect trafficking of NK cells both at steady‐state and during inflammation. S1P_5_, regulated by the expression of the transcription factor T‐BET, is critical for the egress of NK cells from the bone marrow and lymph nodes. NK cells can then return to these tissues via a mechanism that is dependent on CD62L expression.^150^, ^151^, ^152^ Differential gradients of S1P_5_ and the S1P transporter SPNS2 on tissues, particularly lymphatic endothelial cells, combined with CXCR4 expression provide the spatial cues for NK cell localization in tissues.^151^ Although NK cells are found at relatively high frequency in the peripheral blood, they are most frequent in the nonlymphoid organs lung and liver and most abundant in the spleen.^153^, ^154^ A number of factors are necessary for accumulation in tissue‐specific sites. For example, chemokine receptor 1 (CCR1) is necessary for the accumulation of NK cells in the liver.^155^ Thus, multiple organs harbor a significant reservoir of NK cells separate from those found in the blood and under certain physiological conditions such as pregnancy,^156^ or atopic or contact dermatitis,^157^, ^158^ these are massively expanded.

From an evolutionary perspective, the notion that ILCs might only be replenished from local sources would leave the body extremely vulnerable—ILCs would be exposed to depletion by a severe highly acute infection, or more damaging long‐term by a chronic infection without a mechanism to quickly deploy progenitors, or differentiated cells, to replace these cells. Local proliferation could provide some protection, but this is likely to be limited and an infection rapidly outstrip the capacity to generate new cells. Thus, the immune system would be quickly disabled, compromising the mucosal barrier in a life‐threatening manner, and negating the principal role of ILC in maintaining these barriers.

The mechanisms that supports the expansion and contraction of ILCs and their capacity to circulate either at steady‐state or during a response remain contentious and poorly characterized. ILC2 can expand significantly following exposure to an allergen (eg, papain or Aspergillus protease) or the alarmin IL‐33 and these cells produce large amounts of cytokines.^159^ Subsequent to the peak of this response, the number of ILC2 decline but the mechanisms that regulate this reduction are not clear. ILC2, similar to NK cells, express the inhibitory receptors PD‐1 and KLRG1 which perhaps act to regulate this arm of the response.^26^, ^33^, ^67^, ^97^ PD‐1 has been reported in T cells as a mark of immune exhaustion, but separately, this marker can also reflect immune activation. Nevertheless, it highlights an intriguing new regulatory circuitry that appears to be very finely tuned to maintain immune homeostasis requiring substantial more investigation to unravel all the molecular partners involved.

### 3.3 ILC3 are essential to maintain immune homeostasis

ILC3 are highly enriched in the gut mucosal tissues and rapidly respond to the cytokine milieu elicited by the colonization of microbes. Often, we view the role of these cells through the lens of driving immune protection. It is, however, the ability to maintain immune homeostasis that is one of the most fundamental aspects that ensures our health. This requires the capacity of the barrier tissues to continually adjust to unpredictable conditions at those surfaces and to integrate signals from the bacterial communities, epithelial cells, and immune cells. How then do ILCs, particularly ILC3s, participate in orchestrating this type of barrier defense is not well‐understood yet.

LTi cells in the embryo establish the sites at which lymph nodes and mucosal‐associated secondary tissues develop.^16^ These CD4^+^CD3^−^ cells were first discovered in 1997 while a related population termed LTi‐like cells have been identified in the cryptopatches of mice.^160^ This population interacts with B cells to promote IgA production^161^ and the expression of lymphotoxin by ILC3s is critical for both IgA and lymphoid tissue development.^161^, ^162^ Subsequently, LTi and LTi‐like cells have both been identified in murine^98^, ^163^, ^164^, ^165^ and human tissues^166^, ^167^ demonstrating that they are highly conserved between species. In contrast, other ILC3 subsets are scattered along the intestine within the lamina propria where they can expand locally in response to microbial colonization.

Despite the prevalence of ILC3s in the gut, they are not uniformly distributed throughout the entire intestinal tract, being more frequent in the jejunum than the ileum.^141^ This distribution appears to be driven by the heterogeneity of microbes within the intestinal tract which generate metabolites such ligands for aryl hydrocarbon and short chain fatty acids that stimulate ILCs, drive regional specialization and differential distribution. For example, *Lactobacillus*,* Streptococcus,* and *Enterococcus* are localized mainly in the jejunum while segmented filamentous bacteria, Enterobacteriaceae, *Bacteroides,* and *Clostridium* are found lower in the intestinal tract, principally the distal ileum and colon.^141^ Whether microbial stimulation is required for the development of ILC3 still remains contentious. Some studies have demonstrated a paucity of NCR^+^ ILC3 in germ‐free mice^163^, ^164^ while other studies show these populations are preserved.^140^, ^164^, ^168^ However, the ILC populations are significantly amplified by stimulation from microbial communities and the administration of antibiotics eliminates this stimulus and allows the distribution of ILCs to normalize. Expansion of ILC3 appears to depend on the expression of AHR which is induced by tryptophan metabolism to generate indole ligands from the breakdown of glucosinolate glucobrassicin from cruciferous vegetables^169^, ^170^. Recently, the nuclear protein WASH (Wiskott‐Aldrich syndrome protein and SCAR homologue) has been implicated in the recruitment of *Arid1a* to the *Ahr* promoter to activate AHR expression.^171^ This expansion drives the production of IL‐22 by ILC3 which is essential for fucosylation of gut epithelial cells via the induction of the fucosyltransferase, Fut2.^172^ IL‐22 production also promotes the production of the epithelial derived antimicrobial peptide RegIIIγ which is essential for the control of enteric infections such as *Citrobacter rodentium*.^120^, ^169^, ^173^

In *C. rodentium* infection, loss of IL‐22 produced by the NKp46^+^ ILC3 subset does not in itself compromise the capacity to control bacterial colonization as IL‐22 production can be maintained through the NKp46^−^ ILC3 subset.^174^ This raised the notion that innate and adaptive immune cells are highly redundant and challenged our understanding of how overlapping cell types might contribute to maintaining gut homeostasis. The loss of CD4^+^ T cell input, however, resulted in prolonged phosphorylation of STAT3, an activation step that is normally only induced transiently in response to microbial colonization.^175^ Thus, the absence of CD4^+^ T cells uncouples the functionality of NKp46^+^ ILC3 and this cannot be retrieved by sustained IL‐22 secreted by NKp46^−^ ILC3. Instead, it results in impaired host lipid metabolism in the gut. Collectively, these studies demonstrate that sequential interactions between ILC3 and CD4^+^ T cells shape microbial populations allowing the establishment of commensal populations associated with a noninflammatory state and challenged our understanding of how the immune system established this landscape to maintain immune homeostasis.^21^, ^173^, ^174^

## 4 CONCLUSIONS

Elucidation of the key players in the ILC family add an entirely new dimension to how we view the complex interactome necessary for immune protection. ILCs are strategically positioned at all the peripheral and mucosal sites, pivotally positioning them to sense environmental changes and almost immediate responsiveness to any perceived challenges. We are gradually learning the signals that are capable of activating ILCs in autoimmune, allergic and pathogen‐driven responses but still know little about the mechanisms that retain tight control on such pathways to maintain the cells in a quiescent but “alert” state. With the discovery of potential new subsets, intermediate cell types and the gradual emergence of the pathways of ILC plasticity it will be important to understand the cues that allow ILC subsets to adapt to the changing landscape. Those features are drastically different at the beginning of a pathogen or allergen challenge compared with the established setting of an infection or tumor. Although in some cases the transition cell types, for example ILC1 in tumors, seems to disable the function of these cells it is not clear that this would also be true in a pathogen infection or whether pathogens can coopt ILCs to disable their immediate early functions and facilitate pathogen invasion. Mechanisms to replenish ILC in the face of tissue destruction are essential. The current models in which ILC are viewed as relatively static and undergo slow self‐renewal do not appear to fulfill the criteria to ensure that homeostasis would be maintained in the event of a crisis. At mucosal and cutaneous barriers, many insults could easily unravel into highly destructive sequel if multiple avenues are not available to repopulate ILCs. Indeed evidence suggests that ILCs may come from the bone marrow, circulation, local repositioning, or perhaps the thymus, which is emerging as a source of new ILC. We as yet know little about some of these sources, or even how to identify the cells that contribute to the repopulation. This will require a significant shift in our approach to thinking about what progenitors might look like and the circumstances and triggers that might mediate their rapid recruitment to the body's surfaces beyond the finite local tissue reservoirs.

## AUTHOR CONTRIBUTIONS

All authors researched data for the article, made substantial contributions to discussions of the content, wrote the article, and reviewed and/or edited the manuscript before submission.

## CONFLICT OF INTEREST STATEMENT

The authors declare no competing interests.
